# p75 neurotrophin receptor and pro-BDNF promote cell survival and migration in clear cell renal cell carcinoma

**DOI:** 10.18632/oncotarget.8911

**Published:** 2016-04-22

**Authors:** Miguel A. De la Cruz-Morcillo, Julien Berger, Ricardo Sánchez-Prieto, Sofiane Saada, Thomas Naves, Angélique Guillaudeau, Aurélie Perraud, Philippe Sindou, Aurélie Lacroix, Aurélien Descazeaud, Fabrice Lalloué, Marie-Odile Jauberteau

**Affiliations:** ^1^ Limoges University, Equipe Accueil 3842, Cellular Homeostasis and Diseases, Faculty of Medicine, 87025 Limoges, Cedex, France; ^2^ Department of Urology, University Hospital Limoges, 87042 Limoges, Cedex, France; ^3^ PCTCLM/CRIB Unidad de Medicina Molecular Laboratorio de Oncología/Unidad de Biomedicina UCLM-CSIC, Universidad de Castilla la Mancha, 02006 Albacete, Spain; ^4^ Department of Pathology, University Hospital Limoges, 87042 Limoges, Cedex, France

**Keywords:** p75^NTR^, pro-BDNF, TrkB, sortilin, renal cell carcinoma

## Abstract

p75^NTR^, a member of TNF receptor family, is the low affinity receptor common to several mature neurotrophins and the high affinity receptor for pro-neurotrophins. Brain-Derived Neurotrophic Factor (BDNF), a member of neurotrophin family has been described to play an important role in development and progression of several cancers, through its binding to a high affinity tyrosine kinase receptor B (TrkB) and/or p75^NTR^. However, the functions of these two receptors in renal cell carcinoma (RCC) have never been investigated. An overexpression of p75^NTR^, pro-BDNF, and to a lesser extent for TrkB and sortilin, was detected by immunohistochemistry in a cohort of 83 clear cell RCC tumors. p75^NTR^, mainly expressed in tumor tissues, was significantly associated with higher Fuhrman grade in multivariate analysis. In two derived-RCC lines, 786-O and ACHN cells, we demonstrated that pro-BDNF induced cell survival and migration, through p75^NTR^ as provided by p75^NTR^ RNA silencing or blocking anti-p75^NTR^ antibody. This mechanism is independent of TrkB activation as demonstrated by k252a, a tyrosine kinase inhibitor for Trk neurotrophin receptors. Taken together, these data highlight for the first time an important role for p75^NTR^ in renal cancer and indicate a putative novel target therapy in RCC.

## INTRODUCTION

Renal cell carcinoma (RCC) represents 2-3% of all worldwide cancers. Among the different histological types, the clear cell RCC is the most common subtype. Despite earlier diagnosis and considerable progress in treatment of metastatic RCC with targeted therapy, mortality rate remains high. Limited success of the current therapy could be explained by mechanisms of resistance to anti-angiogenic treatment, suggesting that other survival pathways are involved [1]. Thereby, recent advances in understanding the molecular pathogenesis of RCC might provide new therapeutic research pathways [2]. Several studies have highlighted the emerging biological functions of neurotrophins in different cancers [3,4]. However, their expression and role have never been reported in RCC. Therefore, we focused this study on the potential functions of p75^NTR^, a neurotrophin receptor belonging to tumor necrosis factor (TNF) receptor family and its ligand pro-BDNF, in clear cell RCC aggressiveness.

Neurotrophins (NTs) are a family of growth factors initially identified in the nervous system [6] involved in proliferation, differentiation or cell death in several cell types, including malignant cells. These functions depend on their immature or mature forms and the activation of two types of receptors. p75^NTR^, a type 1 transmembrane protein, belonging to the TNF receptor superfamily, is the common receptor for mature NTs and their precursors, the pro-NTs. This receptor is a low affinity receptor for mature NTs and a high affinity receptor for the pro-NTs [5, 6]. The second receptors, neurotrophin tyrosine kinase receptors (NTRK1, 2 or 3) also named tropomyosin kinase (Trk) receptors (TrkA, B, and C), are high affinity receptors for the mature neurotrophins, nerve growth factor (NGF), brain-derived neurotrophic factor (BDNF) and neurotrophin-3 (NT3), respectively. Several studies point out the involvement of BDNF in tumor pathogenesis through TrkB in different cancers [3] such as colon [7, 8], lung [9-11], breast [12] neuroblastoma [13, 14], ovary [15] prostate [16], head and neck [17], as well as lymphoid malignant B cells and myeloma [18-20]. TrkB activation induces phosphorylation of receptor tyrosine residues and signaling pathways through Ras/MAPK (Mitogen-Activated Protein Kinase), PI3K/AKT (Phosphatidylinositol-3 Kinase) and PLC-γ (phospholipase C-γ) activations.

Like other NTs, BDNF is synthetized in a precursor form (pro-BDNF), which is cleaved to form its mature form by proteolytic cleavage [6] through metalloproteinases [21]. Both pro-BDNF and BDNF are secreted and display opposite effects on neural cell proliferation and apoptosis [22] as well as in some malignant cells [7, 18]. The anti-apoptotic function of BDNF is mediated by interactions with the high-affinity receptor TrkB. In contrast, p75^NTR^ binds pro-BDNF, and its ability to induce apoptosis requires its interaction with sortilin (a Vps10p-D, vacuolar protein-sorting domain protein, the third receptor for neurotensin [23]) its co-receptor to form a high-affinity dimeric receptor. p75^NTR^ has not intrinsic catalytic activity and its signaling depends on the recruitment of adaptor proteins [24, 25]. In malignant cells, the role of p75^NTR^ is less well established than for TrkB. Indeed, its overexpression is associated to aggressiveness in human glioblastoma [26, 27], melanoma [28, 29] and breast cancer [12]. In contrast, p75^NTR^ displays a suppressor effect on cell proliferation and invasion in bladder [30], hepatocellular [31] or gastric [32] carcinomas. These opposite roles are related to dual functions of p75^NTR^ in tumor cells, inducing either apoptosis in colorectal and in rat C6 glioma cell lines [7, 27] or cell activation in human breast, glioblastoma and melanoma cancer cells, depending on the cell type and the pro- or mature neurotrophin binding [26, 28, 33].

Although the involvement of BDNF and TrkB remains unknown in renal carcinoma, one study has reported that sera from non-responder patients to Sunitinib (a tyrosine kinase inhibitor with anti-angiogenic activity) contained high levels of BDNF [34]. However, BDNF and receptor expressions in RCC tumors were not described [34]. Therefore, the goal of the present study was to determine the expressions of BDNF and precursor (pro-BDNF) and its receptors in RCC, as well as their biological functions by using RCC-derived cell lines.

We report herein that pro-BDNF and p75^NTR^ are overexpressed in tumors from RCC patients. Only p75^NTR^ correlates with higher Fuhrman grade and worse prognosis in a cohort of 83 clear cell RCC patients. Moreover, p75^NTR^ function was determined in renal cell lines (ACHN and 786-O), showing a clear implication of this receptor in RCC cell survival and migration through pro-BDNF binding.

## RESULTS

### Pro-BDNF and p75^NTR^ expressions are enhanced in renal cell carcinoma tumors

**Figure 1:**
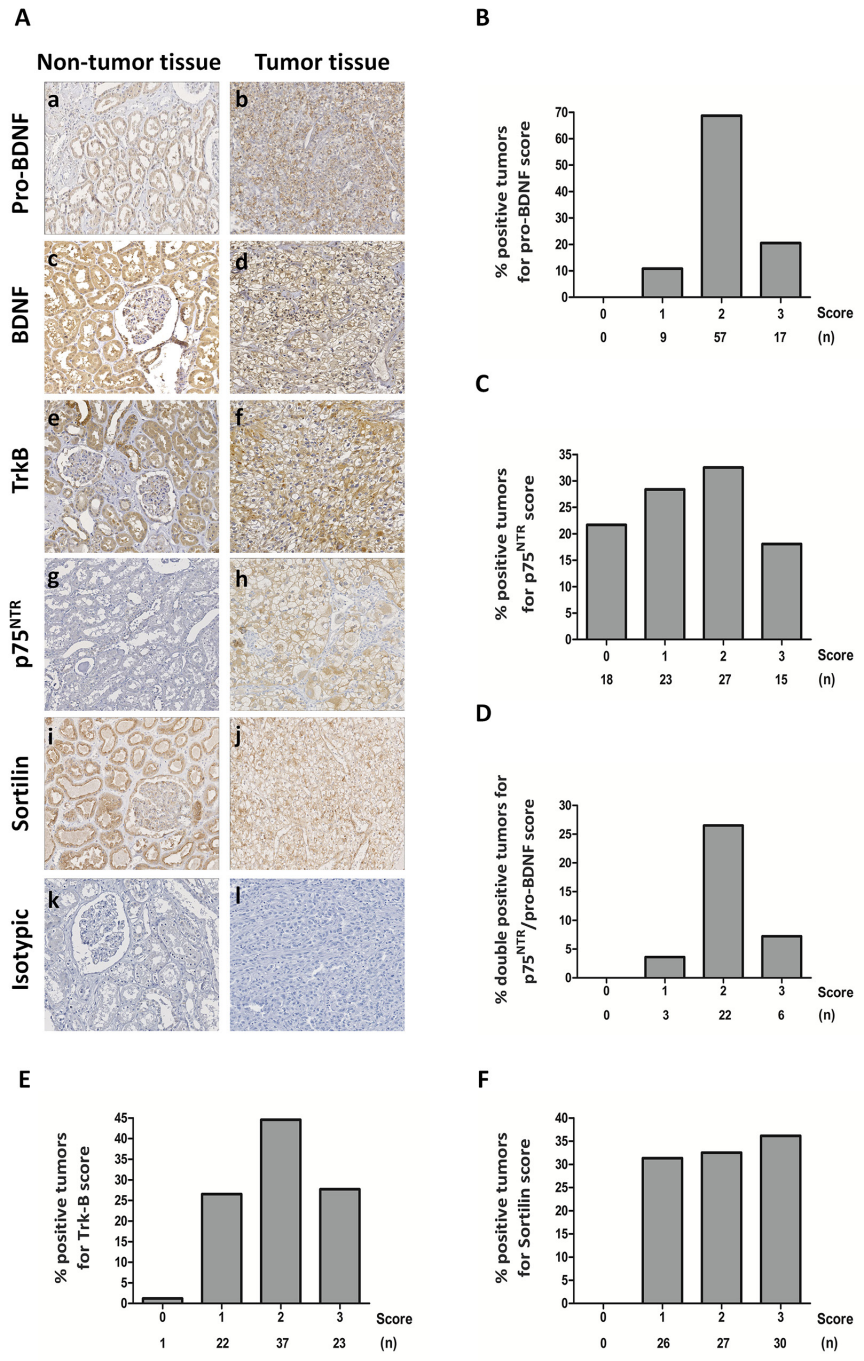
Immunohistochemical staining on clear cell RCC tumors and normal tissues **A.** Tissue Micro Array of normal tissues (a, c, e, g, i, k) and tumor tissues (b, d, f, h, j, l) with immunostaining for pro-BDNF (a, b), BDNF (mature and immature forms recognized by the same antibody) (c, d), TrkB (e, f), p75^NTR^ (g, h), sortilin (i, j) and isotypic controls (k, l). (Magnification 200 x). **B.** Histograms with percentage of positive samples for score of immunostaining intensity (evaluated by image J) for pro-BDNF; **C.** p75^NTR^; **D.** double-positive samples for p75^NTR^ and pro-BDNF; TrkB **E.** and sortilin **F.** (n) indicates the number of cases for each score.

Clear cell RCC tumor tissues from 83 patients (Table 1) who underwent nephrectomy were processed by immunohistochemistry in Tissue Micro Array (TMA) for pro-BDNF/BDNF (both mature and immature forms recognized by a common antibody), pro-BDNF alone, as well as p75^NTR^, TrkB receptor or sortilin. Comparative analyses were performed between normal adjacent tissue and tumor areas by immunohistochemistry for each patient. Various intensity of immunostaining was evaluated by double blind pathologist/assay and also by quantification by Image J software and refereed to the aggressiveness of renal carcinoma (Fuhrman grade). In the absence of available specific antibody to mature BDNF, we performed two immunostaining assays with either a specific anti-pro-BDNF antibody or with an antibody directed against both immature and mature forms of BDNF. Immunostaining assays for pro-BDNF is comparable to results obtained for both mature and immature BDNF indicating that pro-BDNF was highly expressed in most tumor samples as in normal tissues (Figure 1A: a, b and Figure 1B). Whereas pro-BDNF/BDNF, TrkB and sortilin were expressed in normal tissues, mainly in tubules (Figure 1A: a, c, e, i), their expressions were also detected in tumor areas (see Figure 1A). Immunostaining was located in both cytoplasm and membrane for pro-BDNF/whole BDNF (Figure 1A: b and d), mainly in cytoplasm for TrkB (Figure 1A: f) and at the membrane for sortilin (Figure 1A: j). By contrast, p75^NTR^ expression was rarely detected in normal tissues, with a weakly expression in some areas (Figure 1A: g) whereas different levels of expression were detected in some tumor samples (Figure 1A: h), with cytoplasmic and reinforced membranous staining in some tumor areas. The score of immunostaining intensity, evaluated by Image J in the different tumor samples was also indicated for each labelled protein (Figure 1B, 1C, 1E and 1F) as well as double-positive expression for p75^NTR^ and pro-BDNF (Figure 1D). Only p75^NTR^ immunostaining intensity was significantly correlated with symptoms (*P*=0.013), UISS (UCLA Integrated Staging System) prognostic score (*P*=0.040), Fuhrman Grade (*P*=0.001) (Table 2). Multiple linear regression analysis confirmed that changes in p75^NTR^ staining scores were only significantly associated with Fuhrman grade (beta=+0.66, *P*=0.0011), whereas age (*P*=0.17), gender (*P*=0.98), symptoms (*P*=0.78), ECOG (*P*=0.26), T stage (*P*=0.62), N stage (*P*=0.53), M stage (*P*=0.65), pathological stage (*P*=0.76), UISS prognostic groups (*P*=0.31), progression (*P*=0.84), dead (*P*=0.51) or staining scores of pro-BDNF (*P*=0.59), TrkB (*P*=0.80) and sortilin (*P*=0.17) were not found to be independently associated with p75^NTR^ scores.

**Table 1:** Clinical and pathological characteristics of clear cell RCC patients

Clinical characteristics	Mean or n/(%)	Pathological profiles	n/(%)
**Patients**	**n=83**	**T Stage** I	44 (53%)
**Mean Age** (Years)	76.3 (66-89)	II	9 (11%)
		III	30 (36%)
**Gender** (Male/Female)	1.07 (43/40)	**N+ Stage**	4 (5%)
		**M+ Stage**	12 (15%)
**Mean GFR** (MDRD) mL/min	64 (15-156)		
		**Fuhrman Grade** 2	50 (60%)
**Mean Tumor Size (cm)**	5.7 (1.2-18)	3	28 (34%)
		4	5 (6%)
**No Symptom**	41 (49%)		
**Local Symptoms**	28 (34%)	**Pathological Stage** I	43 (52%)
**Systemic Symptoms**	14 (17%)	II	7 (8%)
		III	21 (25%)
**ECOG-PS Score** 1	24 (29%)	IV	12 (15%)
2	32 (39%)		
3	22 (26%)	**UISS** 1	17 (21%)
4	5 (6%)	2	35 (42%)
		3	31 (37%)

**Table 2:** Spearman's Test of correlation between clinico-pathological data and intensity of TMA immunostaining

	Pro-BDNF		p75^NTR^		TrkB		Sortilin	
	*p-value*	*rho*	*p-value*	*rho*	*p-value*	*Rho*	*p-value*	*Rho*
**Gender**	0.893	0.146	0.492	−0.074	0.765	−0.032	0.456	−0.080
**Age**	0.280	−0.117	0.690	−0.043	0.905	0.013	0.895	0.014
**Symptoms**	0.611	0.055	**0.013**	0.263	0.634	0.051	0.068	0.196
**T Stage**	0.305	−0.111	0.149	0.155	0.810	−0.026	0.654	0.048
**N Stage**	0.627	−0.052	0.448	−0.082	0.502	0.073	0.491	−0.074
**M Stage**	0.454	−0.081	0.522	0.069	0.598	0.057	0.845	−0.021
**Pathological Stage**	0.264	−0.120	0.186	0.142	0.770	0.032	0.767	0.032
**UISS Prognostic Group**	0.726	−0.038	**0.040**	0.219	0.662	0.047	0.663	0.047
**Fuhrman Grade**	0.602	−0.056	**0.001**	0.364	0.820	0.025	0.891	−0.015
**Progression**	0.331	−0.107	0.470	0.079	0.673	0.046	0.438	−0.085
**Dead**	0.681	−0.048	0.299	0.120	0.571	0.066	0.479	0.082

### p75^NTR^ and pro-BDNF transcripts are increased in renal cell carcinoma tumors

In order to fully support our previous observation, levels of transcripts for pro-BDNF, TrkB (both full-length and truncated forms) and p75^NTR^ were analyzed by qRT-PCR. To this end, we randomly choose 30 frozen tissues from clear cell RCC tumors. We considered 3 groups according to the *ratio* between tumor tissues and their normal counterparts for each tumor analysis. Lower than 1 (no overexpression), 1-3 fold increase (low overexpression) whereas 3 fold or more increase was considered as high overexpression.

**Figure 2:**
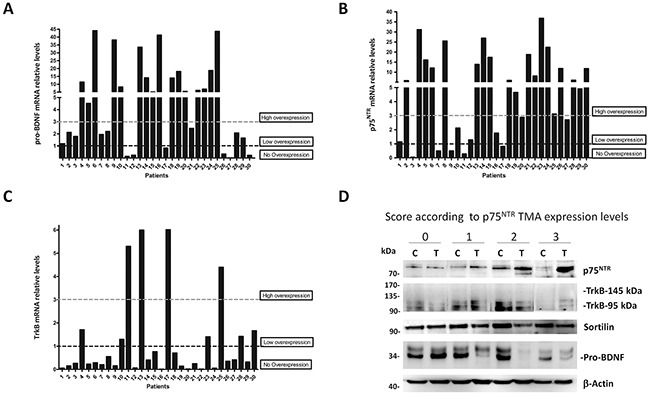
Pro-BDNF, p75^NTR^ and TrkB expressions in clear cell RCC tumors **A.** qRT-PCR analyses of total RNA from 30 tumors and normal kidney tissue patients, expressed in relative mRNA levels from tumor-derived samples referred to their normal counterpart tissue in each case for *pro-BDNF*, **B.** *p75^NTR^*, **C.** *TrkB* (whole forms), *HPRT* was used as housekeeping control. Three groups were defined according to the mRNA ratio between tumor and normal tissues: lower than 1 (no overexpression), 1-3 fold increase (low overexpression) and ≥3 fold increase (high overexpression). **D.** Western blot performed to confirm p75^NTR^, TrkB, sortilin and pro-BDNF protein expressions in tumors (T) and normal counterpart of each tumor sample (C). One tumor sample for each TMA p75^NTR^ immunostaining score (0-1-2-3) was selected to confirm protein levels according to expression levels.

Real time PCR assay showed that 16/30 (53.3%) of the tumors expressed a high level of pro-BDNF transcripts (Figure 2A). In addition, the transcripts for p75^NTR^ were highly overexpressed in 19/30 (63.3%) (Figure 2B). In contrast, those for TrkB (both full-length and truncated forms) were only overexpressed in 4/30 (13.3%) patients (Figure 2C). Interestingly, the pair pro-BDNF/p75^NTR^ appeared overexpressed in more of 50% of analyzed (19 of 30 samples).

To confirm p75^NTR^ protein expression, according to TMA score, we quantified p75^NTR^ levels in immunoblot of protein lysates by choosing a single case per group, in comparison with their normal counterpart tissue (Figure 2D). Results showed a low p75^NTR^ expression in control tissues as well as in score 1 and higher levels in score 2 and 3, as expected by immunostaining analyses. By contrast, western blot confirmed a high basal expression of sortilin, pro-BDNF and TrkB 95 (truncated form) in normal and tumor tissues, in agreement with our observation of Figure 1A.

### Human renal carcinoma 786-O and ACHN cells over-express pro-BDNF, p75^NTR^ and sortilin

Considering our previous results and to study the functions of pro-BDNF, p75^NTR^ and TrkB, in clear cell RCC, two human cell lines derived from RCC were used, a primary renal cell carcinoma (786-O) [35] and a metastatic renal cell carcinoma (ACHN) [36].

**Figure 3:**
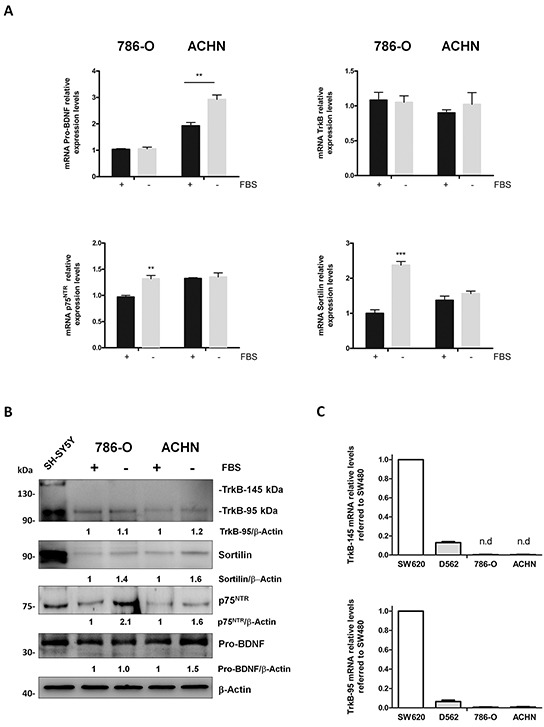
Pro-BDNF, BDNF, p75^NTR^, TrkB and sortilin expressions in 786-O and ACHN renal cell carcinoma cells in presence or absence of fetal bovine serum (FBS) **A.** Relative mRNA levels for pro-BDNF, p75^NTR^, TrkB and sortilin in cell lines cultured in basal conditions (10 % FBS) (black histograms) or in serum deprivation conditions (grey histograms) for 24 hours. (**P<0.01; ***P<0.001). **B.** Protein expressions in same experimental conditions and immunoblotted against indicated antibodies. SHSY5Y neuroblastoma cell line was used as positive control. Values indicate the ratio of relative densitometric values of TrkB-95, sortilin, p75^NTR^ or pro-BDNF compared to β-actin as loading control, normalized to an arbitrary value of 1 obtained in standard cultures containing FBS. **C.** Relative mRNA levels for TrkB-145 (full-length) and TrkB-95 (truncated form) for D562, 786-O and ACHN cell lines compared to colorectal-derived cell line SW620.

Both cell lines expressed pro-BDNF, p75^NTR^, TrkB and sortilin at mRNA (Figure 3A) and protein levels (Figure 3B) with some differences depending on culture conditions including or not FBS in order to mimic stress conditions. Higher levels of pro-BDNF transcripts were detected in ACHN cell line than in 786-O. Besides, in ACHN cells an increase of pro-BDNF levels was detected after 24 hours of serum starvation at mRNA (*P*=0.0012) and protein levels (Figure 3B). p75^NTR^ mRNA increased (*P*=0.001) in starving conditions in 786-O cell line but not in ACHN cells, whereas p75^NTR^ protein levels increased in both serum-free cell lines. Only the truncated form (95 kDa) of TrkB was detected in both cell lines at mRNA and protein levels, without varying expression levels whatever culture conditions (Figure 3A). Finally, only in 786-O cell line, mRNA levels for sortilin increased in starving conditions (*P*=0.0001) while a slight increase of sortilin was detected at protein level for both cell lines (Figure 3B).

Therefore, levels of pro-BDNF and p75^NTR^ increased in both cell lines in starved conditions, whereas low amounts of mature BDNF were detected (data not shown) and TrkB full-length remained almost undetectable (Figure 3B). To verify this observation, we perform a comparative qRT-PCR to determine TrkB-145 and TrkB-95 mRNA levels in different cell lines known to express both forms of TrkB, as observed in our laboratory (Figure 3C). As expected, the colorectal-derived cell line SW480 showed higher levels of TrkB expression (both full-length and truncated isoforms) (Figure 3C) [7]. The other cell line, D562 (head and neck squamous cell carcinoma cell line) had lower levels (Figure 3C). Interestingly 786-O and ACHN cells exhibited the lowest levels for truncated TrkB-95 with undetectable mRNA levels for TrkB full-length, as observed at protein level.

Taken together, these data suggested that p75^NTR^ and pro-BDNF are expressed in our experimental model of renal carcinoma-derived cell lines.

### Pro-BDNF increased cell viability and migration in renal cancer cell lines

**Figure 4:**
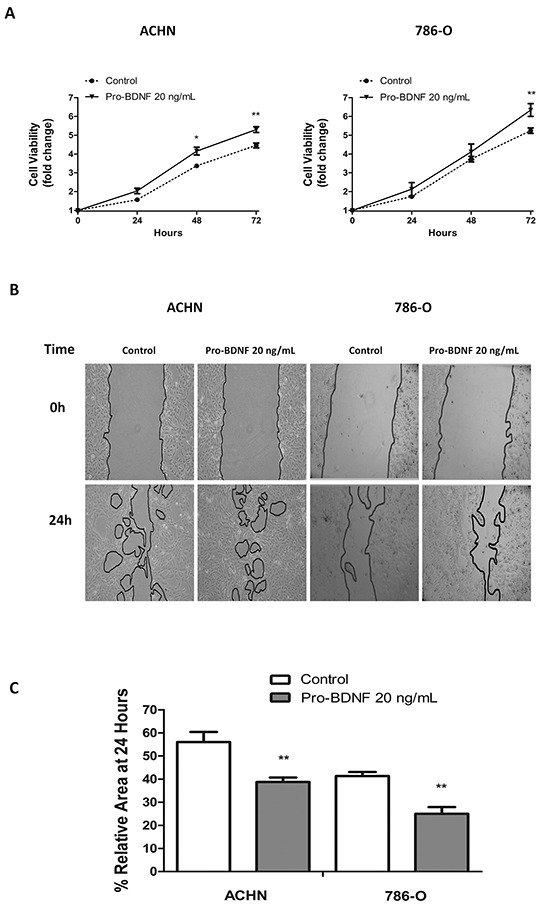
Pro-BDNF increases both cell viability and migration, in ACHN and 786-O renal cell lines **A.** In serum deprivation conditions pro-BDNF (20 ng/mL) was added and cell viability was measured at indicated times by MTT assay for ACHN (left panel) and 786-O (right panel) cell lines. **B.** Wound healing assay was performed in ACHN or 786-O cell lines. Pro-BDNF (20 ng/mL) was added in serum deprivation conditions and migration was evaluated at 24 hours. **C.** Histograms show the area quantification (expressed in percentage) at 24 hours of three independent experiments performed in same conditions for ACHN and 786-0 cells. (**P<0.01).

Therefore, considering all the previous evidences, we have focused the study on the relationship between pro-BDNF/p75^NTR^ and its possible pro-survival role in renal cell carcinoma. Next, the function of pro-BDNF as prosurvival factor through p75^NTR^ was studied in renal carcinoma cell lines. The role of pro-BDNF on cellular viability was evaluated in both cell lines by MTT assay. The viability of both cell lines was increased in the presence of 20 ng/mL of pro-BDNF in serum-free medium (Figure 4A).

Considering that apoptosis induced by pro-BDNF has been previously described in neurons and C6 glioma cells [21, 27], we have evaluated apoptosis in 786-O and ACHN cells in the presence of pro-BDNF (Supplementary Figure S1A). As expected and in contrast to previous reports, pro-BDNF did not induce significant apoptosis in 786-O or ACHN cells. Consistent with these data, cell viability was increased in presence of pro-BDNF alone or combined with GM6001, a broad matrix metalloproteinases (MMPs) inhibitor, used for inhibiting pro-BDNF cleavage. These results confirmed the prosurvival role of pro-BDNF in both cell lines (Supplementary Figure S1B). Interestingly, we have also detected a prosurvival effect for mature BDNF, in both cell lines. (Supplementary Figure S1B).

Then, we search for pro-BDNF function in cell migration by a wound healing assay performed in ACHN and 786-O cells. After 24 hours, a significant migratory response of ACHN (*P*=0.002) and 786-O (*P*=0.0014) cells was observed in presence of pro-BDNF in comparison with control conditions (Figure 4B and 4C). Similar results were obtained in combination with MMP inhibitor, supporting the independent role of pro-BDNF (Supplementary Figure S2A). To validate that results obtained on wound healing assays are not due to cell proliferation, the same experiments were done in presence of cytarabine, (100μM) -a mitotic inhibitor- (Supplementary Figure S2B and S2C). This experiment supports the dual role of pro-BDNF on cell viability and migration in RCC.

### Pro-BDNF promotes cell survival and migration through p75^NTR^ in renal carcinoma cell lines

**Figure 5:**
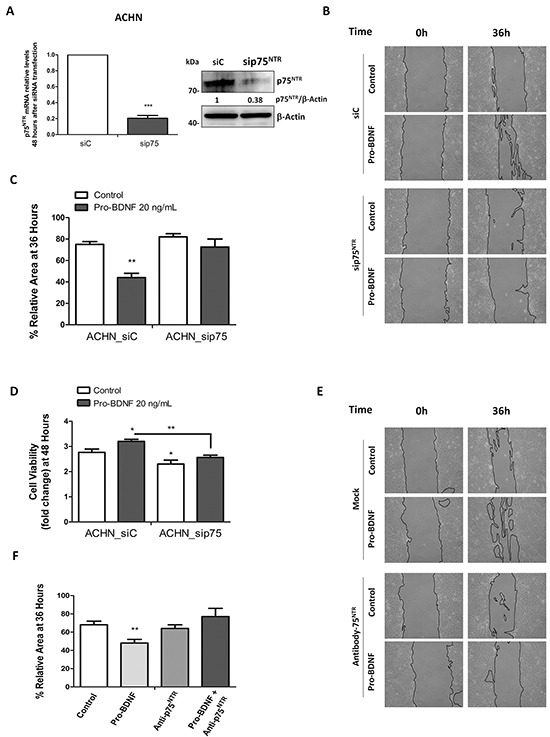
p75^NTR^ RNA silencing or blocking anti-p75^NTR^ antibody inhibits ACHN cell migration **A.** ACHN cells were transfected with control siRNA or p75^NTR^ siRNA, mRNA and protein levels were assessed 48 hours after transfection. **B.** A wound healing assay was performed in serum deprivation conditions for ACHN_siC or ACHN_sip75^NTR^ in absence or presence of pro-BDNF (20 ng/mL) and migration was evaluated at indicated times. **C.** Histogram show the results of 3 independent experiments for wound healing assay under the same experimental conditions that in A. **D.** Viability was evaluated by MTT assay and performed for both, ACHN_siC and ACHN_sip75^NTR^ at indicated times points (in serum deprivation conditions in presence or absence of pro-BDNF as in B). Results are presented as means ± SEM of three independent experiments. **E.** and **F.** Wound healing assay was performed in the presence of a specific blocking antibody for p75^NTR^. Histograms summarize the results of three independent experiments at 36 hours for ACHN cell line.

In order to define the link between p75^NTR^ and the increase of cell viability and migration, we performed siRNA to suppress p75^NTR^ expression. An effective p75^NTR^ knockdown was controlled at protein and RNA levels, 48 hours after transfection in comparison with control siRNA - transfected cells (Figure 5A). Then, wound healing assay was performed with control siRNA and p75^NTR^ - inactivated ACHN cells (Figure 5B). ACHN cell migration was clearly decreased by p75^NTR^ silencing in presence of pro-BDNF (20 ng/mL) in comparison with control siRNA cells (*P*=0.0026) (Figure 5B and 5C). Cell viability was also significantly decreased in silencing-p75^NTR^ cells compared to control *siRNA* in absence of pro-BDNF (*P*=0.035) (Figure 5D). Similar results were observed in presence of pro-BDNF (*P*=0.0066) (Figure 5D). To confirm that p75^NTR^ receptor is essential for ACHN cell migration, same approach was followed by using a specific blocking antibody directed against p75^NTR^ receptor. As expected, at 36 h anti-p75^NTR^ antibody (15 ng/mL) combined with pro-BDNF, decreased the ACHN cell migration, compared to cells treated with pro-BDNF alone (*P*=0.0062) (Figure 5E and 5F).

**Figure 6:**
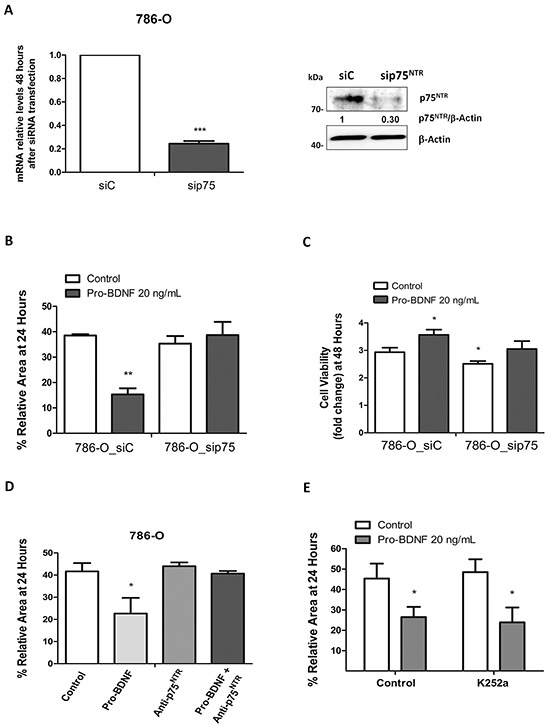
Effects of pro-BDNF on cell viability and migration in 786-O cell line **A.** Interference by siRNA for p75^NTR^ by qRT-PCR and Western blot. **B.** Quantification of three independent experiments for wound healing assay in presence of siC or siRNAp75 (p=0.0053). **C.** Cell viability assay by MTT in 786-O_siC and 786-O_sip75 cells in absence or presence of pro-BDNF at 48 hours. D) Histogram for wound healing assay using a specific blocking antibody (15 ng/mL) for p75^NTR^ that summarize three independent experiments performed at 24 hours (p=0.0484) in identical conditions. **E.** Wound healing assay was performed, with or without, Trk-inhibitor K252a (100 nM) in presence of pro-BDNF. Histogram shows quantification of three independent experiments.

These results strongly suggest the important role for pro-BDNF/p75^NTR^ interaction in the migration of renal carcinoma cells. Likewise, same results were obtained with the 786-O cells. Indeed, p75^NTR^ inactivation by siRNA (Figure 6A) compared to control siRNA, significantly diminished cell migration in presence of pro-BDNF at 24h (*P*=0.0053 *vs* control siRNA cells) (Figure 6B), as well as cell viability (*P*=0.031 at 48 h and *P*=0.001 at 72h) (Figure 6C). As expected, migration of 786-O cells, in presence of pro-BDNF, was inhibited by p75^NTR^ blocking antibody (*P*=0.048 *vs* cells treated with pro-BDNF alone) (Figure 6D). Since Trks family is targeted by k252a [37] and that its combination with pro-BDNF did not modify cell migration, this result fully supports the role of p75^NTR^ on migration independently of Trks receptors (Figure 6E). In sum, we demonstrate that p75^NTR^ inactivation affects both cell viability and migration induced by pro-BDNF in ACHN and 786-O cells, supporting the general feature of our observation.

### Pro-BDNF activates pro-survival signaling pathways

**Figure 7:**
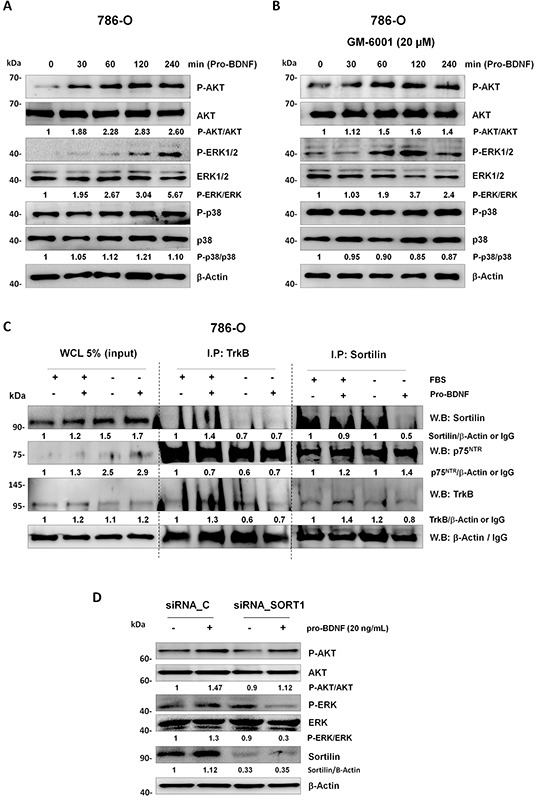
Study of prosurvival signaling pathways and immunoprecipitated receptors complex AKT, ERK1/2 and p38MAPK signaling pathways were analyzed in 786-O cell line cultured in starving conditions. Response to pro-BDNF alone (20 ng/mL) **A.** or with GM6001 (20 μM) **B.** was evaluated at indicated time points. **C.** Immunoprecipitation was performed for TrkB or sortilin in presence or absence or FBS or pro-BDNF (20 ng/mL) as indicated in figure. 5% (v/v) of whole cell lysate (WCL) was used as a control by western blotting in this experiment. **D.** 786-O cell line was transfected with control (siRNA_C) or Sortilin siRNA (siRNA_SORT1). After 24 hours starving 786-O_siC and 786-O_siSortilin cells were treated for one hour with pro-BDNF (20 ng/mL) before AKT and ERK activation studies. Western blots are representative of three independent experiments.

MAPK and AKT activations in response to BDNF have already been reported [38], therefore we analyze MAPKs activation (ERK1/2) and AKT pathways in response to pro-BDNF (20 ng/mL) in 768-0 cells (Figure 7A) alone or in presence of GM6001 MMP inhibitor (20 ng/mL) (Figure 7B and Supplementary Figure S3B). A clear activation of both, AKT and ERK1/2 pathways was detected after pro-BDNF addition to culture media. Conversely, in presence of mature BDNF, no activation of ERK1/2 pathway was detected and a low activation of AKT pathway was detected in presence of BDNF, albeit not comparable to those induced by pro-BDNF (Supplementary Figure S3A). However, no activation of p38MAPK was detected, whatever experimental conditions, supporting the specificity of our observations.

Given that p75^NTR^ can interact with co-receptors, sortilin [39] and TrkB [40], both detected in RCC cells, co-immunoprecipitations were carried out in 786-O cells (Figure 7C). Using anti-sortilin antibody, immunoprecipitated proteins contained high levels of p75^NTR^ associated with truncated TrkB-95, without difference according to protein levels between culture conditions. Interestingly, the interaction of p75^NTR^ with TrkB was also detected by immunoprecipitation with anti-TrkB antibody in the same conditions. Indeed, an immunoprecipitated protein complex containing also high levels of p75^NTR^ associated with TrkB-95 was detected, while sortilin was weakly detected in the complex; whatever experimental conditions (Figure 7C). Consequently, to define the role of sortilin in p75^NTR^ activation through pro-BDNF, we have performed a siRNA sortilin to evaluate AKT and ERK activation in presence of pro-BDNF. Indeed, we observed that AKT and ERK activations were decreased but not abolished in siRNA sortilin cells compared to siRNA control cells (Figure 7D).

Although the precise elucidation of TrkB-95 and sortilin roles in the p75^NTR^ functions remains unknown, our data suggest an interaction between p75^NTR^, sortilin and truncated TrkB 95 that could promote the p75^NTR^ ligand binding.

## DISCUSSION

BDNF and its two main receptors, TrkB and p75^NTR^ have been considered as important factors in oncogenesis. Although the overexpression of TrkB, p75^NTR^ and BDNF have been previously reported in several cancers as poor prognostic factors and to participate to tumor aggressiveness [7, 12, 18, 19], their implication in renal cell carcinoma has not been previously reported. This study highlights the function of p75^NTR^ and pro-BDNF in renal cell carcinoma. Our findings demonstrate that p75^NTR^ and pro-BDNF are overexpressed in RCC patients. Unlike TrkB and sortilin, expressed in normal tissues, p75^NTR^ was only rarely detected in focal areas of normal counterpart tissues. This finding is concordant with previous studies demonstrating that p75^NTR^ expression is sparse and only located in interstitial space and mesenchymal cells in normal adult kidney [41, 42]. Only p75^NTR^ expression in RCC tumors was associated with high Fuhrman grade and correlated to worse prognosis.

At cellular levels, we demonstrate that activation of p75^NTR^ by pro-BDNF plays a major role in cell survival and migration of RCC cell lines, as assessed by transcriptomic and immunological approaches (p75^NTR^ siRNA and blocking anti-p75^NTR^ antibody), through AKT and ERK pathways. This activation is independent of TrkB, as demonstrated by the absence of K252a inhibitory effect on AKT and ERK phosphorylation in presence of pro-BDNF. These findings supported the key function of p75^NTR^ in the aggressiveness of RCC. It was reported that p75^NTR^ exhibits dual functions depending on the presence of a co-receptor and its activation by immature or mature neurotrophins. Opposite effects were initially described in neurons, inducing either apoptosis or cell survival depending on its co-receptor and the release of immature or mature neurotrophins [43]. In neurons, pro-neurotrophins induce apoptosis depending on their binding to p75^NTR^ and its co-receptor sortilin [22, 39], whereas p75^NTR^ induces cell survival through its association to Trk receptors, enhancing the binding of mature neurotrophins [43]. This dual function of p75^NTR^ is also reported in some cancer cells. Indeed, p75^NTR^ suppressed cell proliferation and invasion [30-32] or was associated with cell proliferation and cancer aggressiveness [12, 27, 29, 44]. Concerning TrkB, the other receptor for BDNF, whereas its expression was detected in RCC patients tumors, only the truncated form TrkB-95, known to be deleted for its tyrosine kinase domain [45] was observed in both RCC cell lines, as well as in the proteins extracted from some RCC tumor samples. These results are consistent with a previous study showing that only the truncated form of TrkB is largely expressed in normal human kidney whereas its full-length TrkB-145 is undetectable [46]. Such findings were reported in breast cancer, demonstrating that the association of truncated form of TrkB with p75^NTR^ is essential for ligand binding and p75^NTR^ activation and is not inhibited by K252a [12], a tyrosine kinase inhibitor of Trks [37]. Hence, we have verified that K252a did not suppress cell survival and migration induced by pro-BDNF. The function of p75^NTR^ in cancer aggressiveness was already described in some other cancers. Indeed, high expressions of both pro-BDNF and p75^NTR^ are observed in high grade gliomas with invasive properties [27]. Interestingly, these invasive functions are also dependent of p75^NTR^ and mature neurotrophin binding [26]. Similar function of p75^NTR^ through mature neurotrophin activation was observed in melanoma independently of TrkA [47]. In a similar way, we have found that mature BDNF could also be implicated in cell survival, independently of pro-BDNF, as shown in experiments combining MMP inhibitor. Furthermore, full-length TrkB (145 kDa, containing a tyrosine kinase motif) the receptor for mature BDNF, was not detected at protein and mRNA levels, by contrast to its truncated 95 kDa isoform. These data supported previous studies demonstrating that the association of truncated TrkB to p75^NTR^ enhances the responsiveness to low neurotrophin concentrations [12, 40]. To search for a potential p75^NTR^ co-receptor, immunoprecipitations were performed on RCC cells with its putative co-receptor, TrkB or sortilin. We detected that p75^NTR^ is mainly immunoprecipitated with TrkB-95 and to a lesser with sortilin whatever culture conditions (with or without FBS as well as in presence or absence of pro-BDNF). The sortilin silencing decreased but not abolished AKT and ERK activation in presence of pro-BDNF, suggesting that sortilin facilitates p75^NTR^ function. Therefore, p75^NTR^ activation could be mediated through the complex formed by p75^NTR^, sortilin and TrkB-95. The presence of sortilin in the TrkB/p75^NTR^ complex in RCC cells as described herein was not previously reported in other type of tumor. Its function is not related to the induction of apoptosis as deduced from experiments in the presence of pro-BDNF, inducing cell survival and migration. We have previously described another function of sortilin, as a crucial protein to form a complex with two receptors containing a tyrosine kinase domain, TrkB and EGFR, in lung cancer [48]. Sortilin complexed with TrkA, another tyrosine kinase receptor for NGF, was also identified in breast cancer, promoting cell invasion through pro-NGF binding to this complex [49]. Similar results were reported in melanoma, expressing Trks, p75^NTR^ and sortilin, wherein cell migration and invasion induced by pro-NGF were depending on p75^NTR^ [50]. However, sortilin “*per se*” has been recently described to inhibit cell adhesion in colorectal cancer cell lines [51] and to activate breast cancer cell migration and invasiveness [52]. These mechanisms depend on focal adhesion kinase (FAK) and Src activations, [52, 53] without any change in apoptotic or proliferative cancer cell functions [52]. However, in RCC cells, our results suggest that the role of sortilin is not crucial for p75^NTR^ function through pro-BDNF activation as assessed by the decrease but not suppression of AKT / ERK activation. Therefore, in light of our findings, we postulate that sortilin could associate with p75^NTR^ and truncated TrkB-95. In this complex, p75^NTR^ could be activated by both immature and mature forms of BDNF, while TrkB-95 could enhance the ligand binding to p75^NTR^ as previously described [12, 40].

In conclusion, we report herein that p75^NTR^ expression is correlated with poor prognosis and tumor progression in RCC. The involvement of pro-BDNF and p75^NTR^ in cell survival and migration of RCC cells points out this mechanism supported by p75^NTR^. Our study highlights new mechanisms of aggressiveness in RCC depending on p75^NTR^ and also point to p75^NTR^ as a novel putative target for developing future RCC therapy.

## MATERIALS AND METHODS

### Human RCC immunochemistry in tissue micro array (TMA)

All patients-derived tissues were collected and archived, in Tumor Bank of Limoges University Hospital, under protocols approved by the Institutional Review Board (AC N 2007-34, DC 2008-604, and 72-2011-18). All patients signed an informed consent. Biopsies were obtained from 83 patients who had nephrectomy for RCC between January 2007 and June 2011 at our institution. The following clinico-pathological informations were collected: age, gender, Fuhrman's grade, TNM stage and the UCLA Integrated Staging System (UISS) prognostic scoring. The 2010 TNM system of kidney tumor was used for pathologic staging. For non-metastasis patients, UISS prognostic score was used.

Analysis of 83 tumor biopsies (3 spots per patients) was performed using tissue micro arrays (Superbiochips, Clinisciences), with TSA biotin system kit (PerkinElmer), according to the manufacturer's instructions. We exclude high necrosis and inflammatory areas. After pretreatment of slide, they were dewaxed, rehydrated (Toluene, alcohol, PBS), and incubated in citrate buffer at pH 7 (200 μM citric acid and 9.8 mM sodium citrate) to reveal cytoplasmic antigen. After inhibition of endogenous peroxidase, slides were incubated with following antibodies: rabbit polyclonal antibody directed against internal region of human BDNF common to mature BDNF and its precursor pro-BDNF (Santa Cruz, 546 N-20) or with specific rabbit polyclonal anti-pro-BDNF antibody specific to the pro domain region of human pro-BDNF (Alomone, ANT-006). The corresponding receptors were studied with rabbit anti-p75^NTR^ (Santa Cruz Biotechnology, 8317 H-137), anti-TrkB antibodies (Santa Cruz, 8316 H-181) or anti-sortilin (Santa Cruz, 30217 H-300) all of them diluted at 1/100 in blocking buffer, overnight at 4°C. After 3 washes with PBS, slides were incubated with a secondary anti-rabbit immunoglobulin antibody (K4011, Dakocytomation) for 1 hour. Slides were rinsed with PBS, incubated with diaminobenzidine (DAB) and counterstained with hematoxyline. Slides are coverslipped using Eukit mounting medium (03989, Fluka). Negative control slides were done with the secondary anti-rabbit immunoglobulin by exclusion of the primary antibodies. Slides were observed using a Leica light microscope and Axivision software. Immuno-reactivity was quantified by two different methods: a) independent observers (J.B and A.G) including a pathologist in double blind. The levels of staining were scored based on staining intensity within the tumor sample as 0: no staining, 1: weak staining, 2: moderate staining and 3: strong staining, and compared to clinical pathological data; or b) using Image J software (ImageJ 1.48v National Institutes of Health, USA). In this case pictures were taken considering the same ROI (Region of interest) area. The quantification of immuno-staining was performed by using color deconvolution function (for H-DAB function). Then the immunostaining area was measured in arbitrary area units (pixels^2^) and expressed as the percentage of total ROI area: Arbitrarily Scores were considered as: Score 0: 0-1.5% Score 1: 1.5-15%. Score 2: 15-30%. Score 3: >30% over total image area.

### RNA isolation, reverse transcription and real-time quantitative PCR

Quantitative RT-PCR was performed for selected 30 patients who had nephrectomy for RCC between 2007 and 2009. For each patients, 15 g of normal and tumor frozen tissues were obtained and then were be crushed by Mixer Mill 300 (Bertin Technologies). Total RNA was extracted using RNeasy Mini kits (QIAGEN). Reverse transcription was performed from 1 μg total RNA using (Superscript III first strand kit, Invitrogen). Specific primer pairs (Applied Biosystems) corresponding to TrkB (Full-length and truncated TrkB-95 isoforms), p75^NTR^ and pro-BDNF were used. Then, real-time PCR was performed on thermocycler Applied 7500 Fast (Roche Applied Science, Indianapolis). Data were normalized based on HPRT gene transcript levels. For RCC cell lines were cultured in standard and starving conditions. Total RNA was extracted as described before. RNA quantity and quality were assessed by measuring the absorbance using the nanodrop spectrophotometer ND-1000 (Labtech). First-strand cDNA synthesis was generated by using Superscript III (Invitrogen). PCR was performed using Taq DNA polymerase (Invitrogen) and specific primers for whole BDNF transcript, TrkB (Full-length and truncated isoform), p75^NTR^, GAPDH and HPRT as housekeeping controls. In all cases quantification was performed by the comparative cycle threshold method using the HPRT or GAPDH RNA expression level as internal control [54]. Primers used in this study were previously described [7].

### Cell lines

Two Renal carcinoma cell lines were used. A primary clear cell renal adenocarcinoma (786-O) [34] and a metastatic (pleural effusion) renal cell adenocarcinoma (ACHN) [35] were purchased from American Type Culture Collection (ATCC) (LGC Standards SLU, Barcelona, Spain). Cells were maintained in Eagle's Minimum Essential *Medium* (EMEM) at 37°C in a 5% CO_2_ atmosphere. EMEM was supplemented with 10% fetal bovine serum (FBS), 300 mg/L of L-glutamine, penicillin (100 units/mL) and streptomycin (0.1 mg/mL) (Thermo-fisher scientific). For functional assays, some experiments were also performed in serum starved EMEM, to search for an enhanced expressions of p75^NTR^ and pro-BDNF as indicated in figures and text.

### Antibodies and reagents for cell studies

Western blotting was performed with specific antibodies against phospho-p38MAPK-T180/Y182 (#4511), or phospho-AKT-T308 (#2965) from Cell Signaling, or phospho-ERK1(T202/Y204)/ERK2(T185/Y187) (R&D AF1018) and ERK1/2 (AF1576) were obtained from R&D. Antibodies against whole form of p38MAPK (#8690), and AKT (#4691) were from Cell Signaling. p75^NTR^ (Santa Cruz, sc-8317) or (Abcam, ab52987). Pro-BDNF antibody was purchased from Alomone (Cat#: ANT-006). Trk-B (#610101) and sortilin (#612101) were from BD company. As loading control β-actin (Sigma-Aldrich A5441) was used. Functional assays were realized with specific antagonistic antibody to block p75^NTR^ (Upstate, clone ME 20.4, cat# 05-446) used at 15 ng/mL.

In some experiments, we used a broad-spectrum inhibitor of matrix metalloproteinases (MMPs) GM6001 (Calbiochem, CAS142880-36-2) (20 μM) to inhibit the cleavage of pro-BNDF in mature BDNF [21]. In all cases, GM6001 was added to culture media 30 minutes before pro-BDNF treatment.

### Transfections and RNA interference assays

For interference assays, ACHN and 786-O cell lines were seeded in 6-well plates. When cultured cell lines reached optimal confluence conditions were transiently transfected by using INTERFERin, following manufacturer's instructions (Polyplus transfection). 20 ng of siRNA against p75^NTR^ (ON-TARGET plus Human NGFR L-009340-00-0005), or siRNA control (ON-TARGET plus Non-targeting pool D-001810-10-05), or siRNA Sort1 (ON-TARGET plus Human SORT1 siRNA L-010620-00-0005) (Thermo Scientific-Dharmacon), were used in each transfected condition. Then, 48 hours after transfection cells were starved and treated for experimental conditions as described above. For validation of experimental conditions, p75^NTR^ and sortilin levels were evaluated by RT-PCR and western blot assay. Each transfection experiment was repeated at least thrice.

### Western blotting

Proteins were obtained from cell lysates of culture cell both in normal and deprived serum condition. After two washes in PBS, cells lysates were collected in lysis buffer (25 mM HEPES pH 7.5, 0.3 M NaCl, 1.5 mM MgCl2, 0.2 mM EDTA, 1% Triton X-100, 0.1% SDS, 0.5% Deoxycholic Acid, 20 mM B-glycerophospate) plus protease and phosphatase inhibitors (2 μg/mL Leupeptin, 2 μg/mL Aprotinin, 1 mM PMSF and 0.1 mM Na3VO4). Then 30 μg of proteins were loaded on 8-12% SDS-PAGE. Proteins were transferred on activated PVDF membrane and blotted against the indicated antibodies. Antigen detection was achieved by enhanced chemiluminescence (ECL, Amersham). All Western blots detection was performed using a G:Box detector (Syngene). Protein quantification was performed using BCA Protein Assay Kit (Pierce) following manufacturer's instructions. Results show a representative blot out of three experiments with similar results. Images were quantified by using Image J software.

### Immunoprecipitation

Cells were solubilized in 500 μL of Triton X-100 immunoprecipitation buffer containing: 50 mM Tris-HCL pH 7.5, 150 mM NaCl and 2 mM EDTA solution. Protein A sepharose from Staphylococcus-aureus 10% v/v (50 μL per condition) was added, and samples were incubated for 1 hour at 4°C. Samples were centrifuged for 15 sec and supernatant was transferred to a fresh 1.5 mL tube. For immunoprecipitation assay, antibodies from BD company against TrkB (#610101) and sortilin (#612101) were used along with protein A Sepharose (50 μL), and samples were incubated over-night at 4°C. Samples were centrifuged for 15 sec to pellet protein A Sepharose and supernatant was discarded. Pellets were washed three times with immunoprecipitation buffer. Finally, pellets were resuspended in 30 μL of SDS-PAGE sample buffer containing DTT (50 mM) and analyzed by SDS-PAGE as indicated previously in western blotting section. An aliquot of total whole cell lysate, 5% (v/v) was used for western blot (input) as an internal control.

### Viability assays

Sub-confluent monolayer cultures were trypsinized, and cells were plated in 96-well plates at a density of 2.500 cells per well. Cell viability was analyzed at 1, 2, 3 days by MTT-based assay. Briefly, MTT at 0.5 mg/mL was added to the medium in each well and plates were returned to the incubator for 1 h. The medium-MTT was then removed, 100 μL of DMSO was added to each well, and the plate was kept in agitation for 5 min in the dark to dissolve the MTT-formazan crystals. The absorbance of the samples was then recorded at 570 nm. Four wells were analyzed for each condition, and wells containing medium plus MTT but no cells were used as blanks. Pro-BDNF was used at 20 ng/mL.

### Apoptosis assay

Apoptosis was evaluated by measurement of cytoplasmic soluble nucleosome determined following the manufacturer's instructions of ELISA Cell Death kit (Roche). ACHN cells were seeded in 96-multiwell plate (5000 cells/well) and cultured for 24 hours. After this time, 20 ng/mL recombinant human pro-BDNF (Cat# B-257), 10 ng/mL of mature BDNF (Cat# B-250) (Alomone) or PBS as control were added to ACHN cells.

In indicated cases, GM6001 or neutralizing anti-BDNF antibody was also used alone or in combination with pro-BDNF or BDNF. Absorbance values were measured at 405 nm with an ELISA reader (Labsystems). The absorbance obtained was normalized to a value of 1, corresponding to control (ACHN cells in no serum culture conditions) as previously described [18, 55]. Each experiment was performed in triplicate for at least three times independently.

### Wound healing assays

To perform wound healing assays, ACHN and 786-O cells were growth to confluence (>90%) in 6-well dishes. A small area was then disrupted by scratching the monolayer with a 100 μL plastic pipette tip. After scratching, wells were washed wit PBS and cells were cultured in starving conditions and treated as indicated in antibodies, chemicals and treatments section. Cells were inspected microscopically every 6 hours. Image J software (NIH, Bethesda, MD, USA) was used to calculate area and migration distance of the cells. Every point was referred to itself control at time 0 hours for the representation and statistical study. For experimental use, pro-BDNF was used at 20 ng/mL, and specific anti-p75^NTR^ blocking antibody was used at 15 ng/mL.

### Statistical analysis

Statistical analysis for TMA was performed with logistic regression analysis to correlate clinical data and the score of immunostaining intensity for p75^NTR^, sortilin, TrkB and pro-BDNF (Statview 5.0, SAS Institute Inc., Cary, NC). Multiple linear regression was used to assess whether age, gender, symptoms, ECOG, T stage, N Stage, M stage, pathological stage, UISS Prognostic group, Fuhrman grade, progression, dead or staining score (Pro-BDNF, TrkB and sortilin) were associated with score of p75^NTR^ (Statview 5.0). Differences were considered significant at *P* <0.05. The comparison of cell lines was determined by Student's *t* -test (Statview 5.0). All results are represented as mean ± standard error of the mean (SEM) of at least three independent experiments performed in triplicate.

## SUPPLEMENTARY FIGURES


