# Supplementary material for: p75 neurotrophin receptor and pro-BDNF promote cell survival and migration in clear cell renal cell carcinoma
Source: Oncotarget. 2016 Apr 22;7(23):34480–97. doi: 10.18632/oncotarget.8911 (PMC5085170; doi:10.18632/oncotarget.8911)
Supplement: Supplementary file 1 [file oncotarget-07-34480-s001.pdf]

p75 neurotrophin receptor and pro-BDNF promote cell survival and migration in clear cell renal cell carcinoma

SUPPLEMENTARY FIGURES

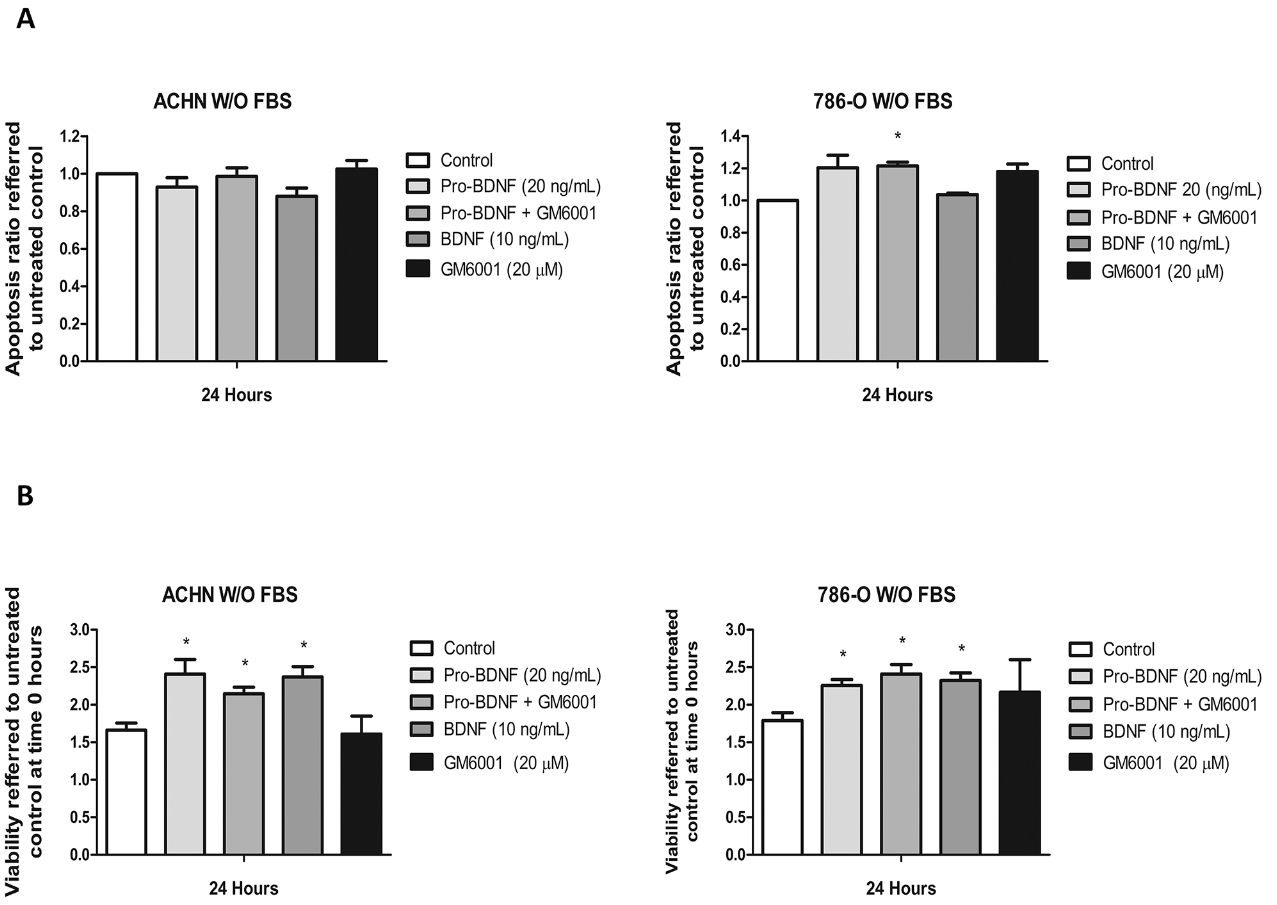

Supplementary Figure S1: Study of apoptosis/viability in ACHN and 786-O renal cell lines. A. To study the apoptotic response in ACHN and 786-O cell lines, a specific kit was used (Cell Death Detection ELISA PLUS Cat.No.1-774-425) following manufacturer's instructions. Without (W/O) FBS culture conditions, apoptosis was evaluated in response to Pro-BDNF (20 ng/mL) or BDNF (10 ng/mL). GM6001, a broad-spectrum inhibitor of MMPs (Calbiochem CAS142880-36-2) was used in presence of Pro-BDNF or alone, as indicated in figure. **B.** In parallel, viability was evaluated under identical experimental conditions by using XTT (Roche Ref 11-465-015-001) to validate our results. Results are represented as mean ± SEM of at least three independent experiments performed in triplicate. Differences were considered to be significant at *p<0.05 or level.

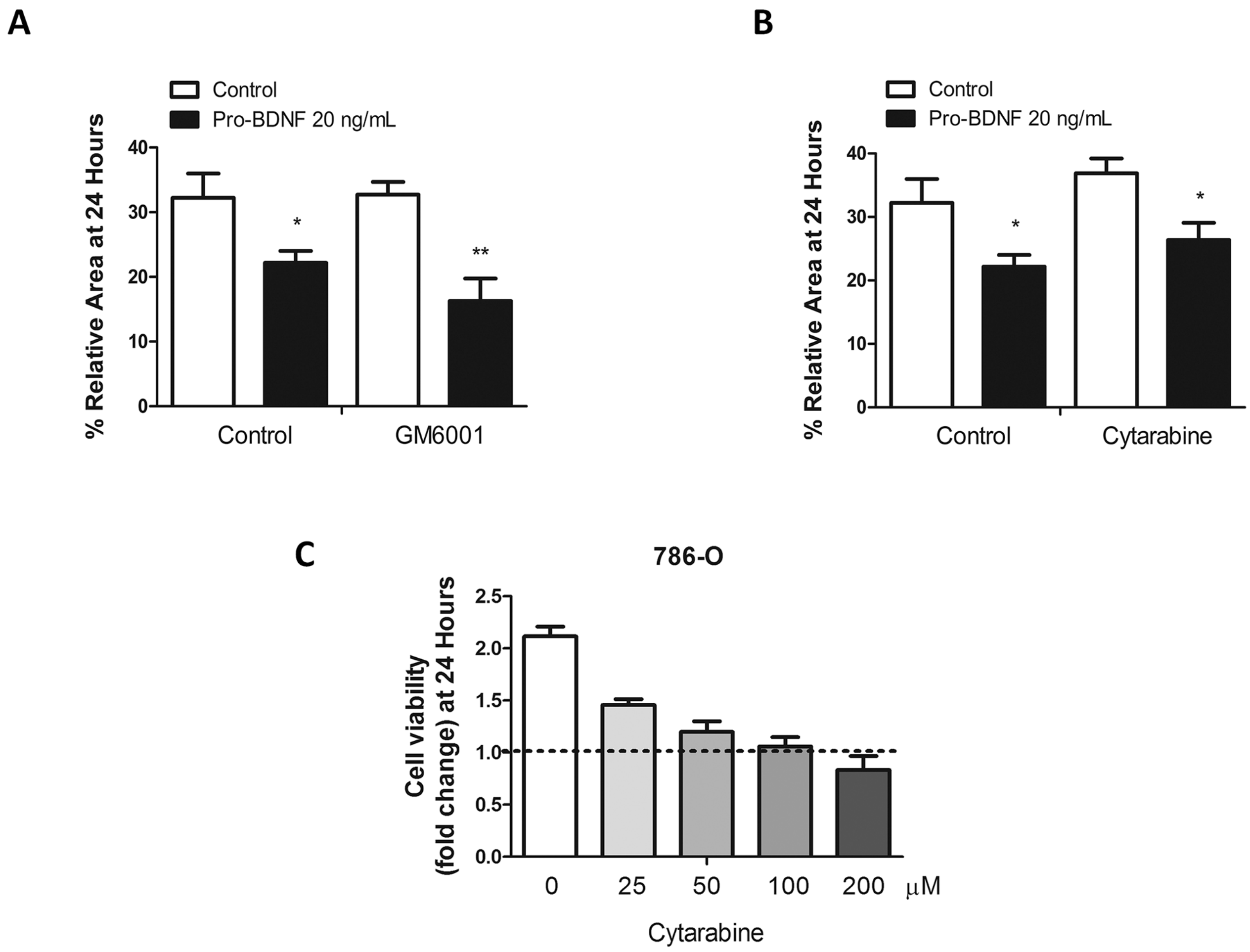

Supplementary Figure S2: Wound healing assay in 786-O cell line in presence of GM6001 and cytarabine. Histograms show the area quantification (expressed in percentage) of three independent experiments (wound healing assay) performed in 786-0 cell line. Cells were pretreated with GM6001 (20 μM) **A.** or cytarabine (100 μM) **B.** After 30 minutes, pro-BDNF (20 ng/mL) was added to culture media (in serum deprivation conditions), and migration was evaluated at 24 hours. **C.** Viability at 24 hours was evaluated by XTT (Roche Ref 11-465-015-001) in 786-O cell line in presence of Cytarabine (Sigma-aldrich, European Pharmacopeia -EP- C3350000) at indicate doses. Results are represented as mean ± SEM of at least three independent experiments performed in triplicate. Differences were considered to be significant at *$p<0.05$ or **$p<0.01$ level.

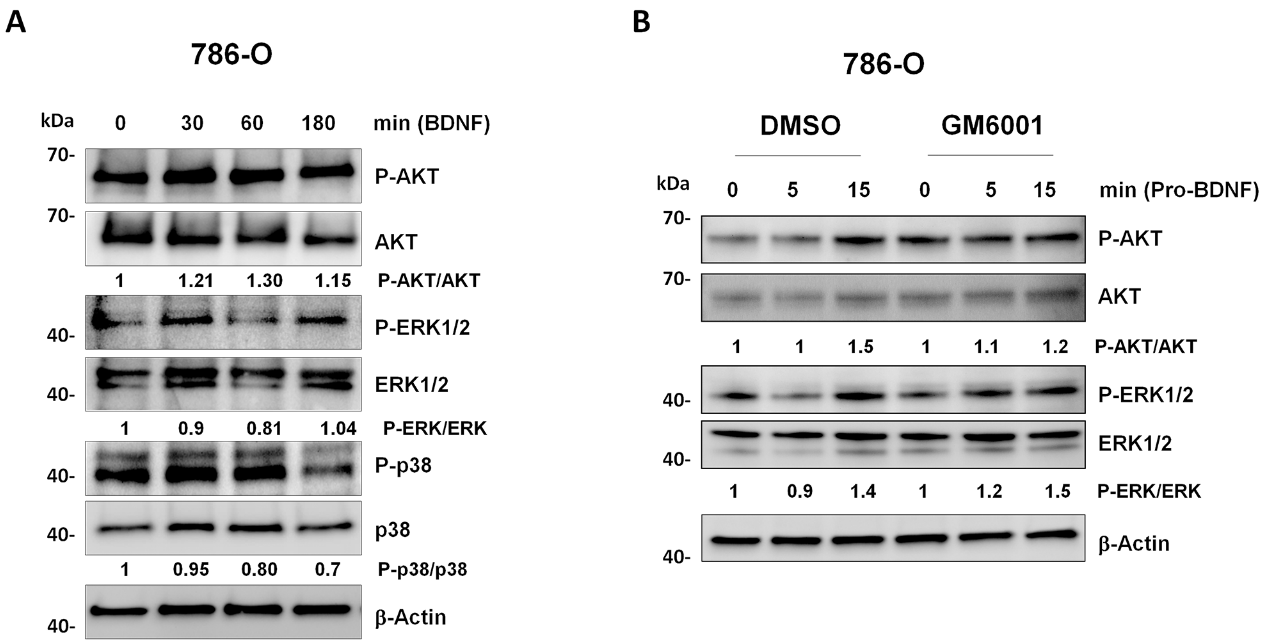

Supplementary Figure S3: Study signaling pathways in 786-O cell line. A. P-AKT, P-ERK1/2 and P-p38MAPK signaling pathways were analyzed in 786-O cell line cultured in starving conditions. Response to BDNF (10 ng/mL) was evaluated at indicated time points. **B.** P-AKT and P-ERK pathways were analyzed at indicates times, in presence of Pro-BDNF (15 ng/mL) alone or with GM6001 (20 μM). Cells were treated with DMSO (control) or GM6001 30 minutes before Pro-BDNF addition. Western blots are representative of three independent experiments.
